# Outcomes of a single site clinic for eating disorders in child and youth mental health

**DOI:** 10.1186/2050-2974-3-S1-O7

**Published:** 2015-11-23

**Authors:** Salvatore Catania, Richard Litster, Tania Withington

**Affiliations:** 1 Child and Youth Mental Health Service, Queensland Children's Hospital, Brisbane, Australia

## 

Anorexia Nervosa (AN) and restrictive eating disorders present a significant burden of disease within the Australian Community with AN having the highest mortality rate of all psychiatric disorders. Family Based Treatment for Adolescent Anorexia Nervosa (FBT) has the best evidence worldwide for successful treatment outcome and sustained recovery.

In October 2012, The Children Health Services District established a specialised weekly clinic to provide FBT for community based treatment of AN. Multidisciplinary staff with a background in Family therapy were trained in FBT. The aim was to develop a specialist service targeted at a high risk and resource-intensive population.

Prior to establishing the clinic in this form Family based Treatment was provided at three separate community clinics. The intention of establishing a single clinic was to facilitate greater access to supervision and increase model fidelity, increase the capacity of the service, enhance the sense of team cohesion and increase the overall number of referrals.

The presentation will outline the outcomes from the first two years of this specialist clinic. These include, a reduction in length of stay in hospital inpatient units, an increased proportion of families completing FBT, reduced length of time in treatment and an increased identification of anorexia nervosa with corresponding increased referrals into FBT.

